# Integrative analysis of vitamin D, ferritin, and eosinophilic inflammation in predicting acute exacerbations of childhood asthma

**DOI:** 10.3389/fimmu.2026.1746377

**Published:** 2026-04-20

**Authors:** Pingping Wang, Jin Yao, Yaqiong Li, Zhanjun Zhang, Xiaofei Jin, Li Li, Xiaorong Huang

**Affiliations:** 1 Department of Medical Laboratory, Luoyang Maternal and Child Health Hospital, Luoyang, Henan, China; 2 Department of Infection and Public Health Management, The Second Affiliated Hospital of Henan University of Science and Technology, Luoyang, Henan, China; 3 Medical Imaging Department, Luoyang Maternal and Child Health Hospital, Luoyang, Henan, China; 4 Department of Pathology, Luoyang Maternal and Child Health Hospital, Luoyang, Henan, China; 5 School of Law and Economics, Wuhan University of Science and Technology, Wuhan, Hubei, China; 6 Luoyang Research Center for Inheritance and Innovation of Chinese Historical Civilization, Luoyang Institute of Science and Technology School of Marxism (LIT), Luoyang, Henan, China

**Keywords:** acute exacerbation, decision curve analysis, eosinophilic inflammation, ferritin, logistic regression, pediatric asthma, vitamin D

## Abstract

**Background:**

Vitamin D and iron metabolism are increasingly recognized as potential modulators of airway inflammation, yet their interrelationship in pediatric asthma remains unclear. This study investigated the associations of serum vitamin D, ferritin, and eosinophilic inflammation with acute asthma exacerbations in children and explored their potential interaction.

**Methods:**

This single-center retrospective study included 120 children with asthma, comprising 60 with acute exacerbation and 60 in clinical remission. Serum 25-hydroxyvitamin D [25(OH)D], ferritin, interleukin-6 (IL-6), and eosinophil-related indices were measured. Group comparisons, Spearman correlation analysis, univariate and multivariable logistic regression, restricted cubic spline analysis, and decision curve analysis were performed.

**Results:**

Compared with the remission group, children with acute exacerbation had significantly higher ferritin levels (median 145 vs. 82 ng/mL, P < 0.001) and eosinophil percentage (6.0% vs. 4.7%, P < 0.001), but lower vitamin D levels (18.6 ± 7.2 vs. 24.3 ± 8.7 ng/mL, P = 0.021). In multivariable logistic regression, ferritin (OR = 1.13, 95% CI 1.07–1.18) and eosinophil percentage (OR = 2.01, 95% CI 1.34–2.70) remained independently associated with acute exacerbation, whereas vitamin D was not statistically significant after adjustment, although the association remained directionally inverse (OR = 0.92, 95% CI 0.84–1.02). No significant interaction between ferritin and vitamin D was observed, but interaction testing was limited by sample size. Restricted cubic spline analysis suggested an inverse linear association between vitamin D level and exacerbation risk. The combined model including ferritin, eosinophil percentage, and vitamin D showed high apparent discrimination (AUC = 0.973, 95% CI 0.952–0.994), although this finding should be interpreted cautiously because of overfitting risk.

**Conclusions:**

Ferritin and eosinophil percentage were independent risk factors for acute asthma exacerbation in children. Vitamin D showed an inverse association in unadjusted and dose-response analyses but was not an independent predictor after multivariable adjustment. These findings support a possible link between metabolic and inflammatory pathways in childhood asthma, but larger studies are needed for validation.

## 1 Introduction

Asthma is one of the most common chronic diseases in children worldwide, affecting approximately 262 million individuals and causing more than 400,000 deaths annually (1). Despite continuous advances in pharmacological treatment, childhood asthma remains a leading cause of emergency department visits and hospitalizations. Acute exacerbations not only impose a substantial disease burden but also accelerate airway remodeling, impair lung function development, and significantly reduce quality of life (2). Recent studies have shown that childhood asthma is not a single disease but a syndrome comprising multiple inflammatory endotypes, the most representative of which are the type 2-high (T2-high) eosinophilic and neutrophilic inflammatory phenotypes (3, 4). Therefore, identifying biomarkers that can reflect immune activation status and predict the risk of acute exacerbations is of great importance for precision prevention and stratified management.

Among various environmental and nutritional factors, vitamin D has attracted considerable attention because of its potential immunomodulatory effects (5). Its active metabolite, 1,25-dihydroxyvitamin D_3_, is thought to participate in immune regulation by modulating Treg function, Th2-related cytokine release, and antimicrobial peptide expression; however, the clinical significance of these mechanisms in acute exacerbations of childhood asthma has not been fully elucidated (6, 7). Multiple observational studies have reported that low serum 25-hydroxyvitamin D [25(OH)D] levels are associated with increased airway hyperresponsiveness, elevated IgE levels, and higher frequency of asthma exacerbations (7, 8). Nevertheless, observational associations do not equate to independent causal effects, and the results are susceptible to confounding by medication use, sunlight exposure, dietary intake, and underlying inflammatory status. Evidence regarding whether vitamin D supplementation can consistently improve outcomes in childhood asthma remains heterogeneous. Results from large-scale clinical trials and meta-analyses are inconsistent, and some studies have found no significant benefit of vitamin D supplementation on asthma control (9, 10). These discrepancies may be related to genetic, ethnic, and environmental differences, as well as inadequately evaluated interactions with metabolic pathways (such as iron metabolism and inflammatory responses) (11).

In recent years, iron metabolism has been increasingly recognized as an important regulator of immunity and oxidative stress and is closely associated with chronic airway diseases (12). Ferritin is not only an intracellular iron-storage protein but also an acute-phase reactant that reflects systemic inflammatory status (13). Elevated serum ferritin is commonly observed in conditions accompanied by oxidative stress and cytokine activation, including allergic airway inflammation (14). Interleukin-6 (IL-6), a key inflammatory mediator in acute asthma exacerbations, can promote hepatic hepcidin synthesis, thereby reducing bioavailable iron and forming a positive-feedback loop between inflammation and iron metabolism dysregulation (15). The IL-6–hepcidin axis influences systemic iron availability by restricting iron efflux, providing a biological basis for the link between inflammation and iron metabolic disorders. Previous studies have shown that vitamin D can downregulate hepcidin expression and affect iron transport (16), suggesting that vitamin D deficiency may promote inflammatory responses by altering iron metabolism. However, although airway iron homeostasis abnormalities have been observed in patients with asthma or recurrent wheezing, the supporting clinical evidence remains limited, particularly in the setting of acute exacerbations in children, where systematic evaluations are still lacking. To date, there is no comprehensive study examining the interrelationships among vitamin D status, iron metabolism, and eosinophilic inflammation in childhood asthma.

Emerging research indicates that single biomarkers often fail to accurately capture complex immune-metabolic networks, and integrated multi-pathway analyses may better predict clinical outcomes (17, 18). Nevertheless, few studies have simultaneously integrated vitamin D, ferritin, and eosinophilic indices to construct a composite model for assessing the risk of acute asthma exacerbations. Furthermore, it remains unclear whether vitamin D status exhibits interactive patterns with iron metabolism indices or eosinophilic inflammation in the context of exacerbation risk. Given that vitamin D deficiency, inflammatory activation, and iron homeostasis alterations may coexist in chronic airway diseases, a combined evaluation of these indicators may help provide a more comprehensive understanding of the immune-metabolic characteristics associated with acute exacerbations of childhood asthma.

This study retrospectively analyzed 120 children with asthma, stratified into an acute exacerbation group and a clinical remission group according to disease status. We compared differences in hematological, inflammatory, immunological, and nutritional-metabolic indices between the two groups, with a particular focus on the predictive roles of serum ferritin (Ferritin), eosinophil percentage (EOS%), and 25(OH)D levels in acute exacerbations. A multivariable logistic regression model incorporating these indices was constructed. Restricted cubic spline (RCS) analysis and decision curve analysis (DCA) were also applied to evaluate dose–response relationships and the clinical utility of the model. We hypothesized that elevated Ferritin and EOS% would be associated with increased risk of acute asthma exacerbations, whereas 25(OH)D levels would show an inverse association. We further explored potential interaction patterns between vitamin D status and iron metabolism/eosinophilic inflammation indices. The aim of this study was to investigate, from an integrated immune-metabolic perspective, the relationships among vitamin D, ferritin, eosinophilic inflammation markers, and acute exacerbations of childhood asthma, thereby providing preliminary evidence for subsequent larger-scale studies.

## 2 Methods

### 2.1 Study population

This study was a single-center retrospective analysis. Clinical data were collected from children diagnosed with asthma at the Luoyang Maternal and Child Health Care Hospital (a tertiary A-level maternal and child health hospital) between January 2023 and December 2024, resulting in the enrollment of 120 children. Sample size was estimated *a priori* assuming a baseline area under the curve (AUC) of 0.7 for a single biomarker, with an anticipated model improvement of ΔAUC = 0.05, α = 0.05, and power (1–β) = 0.80; approximate calculations using the Hanley–McNeil formula indicated that approximately 60 participants per group would be required. The sample size in this study was primarily determined for exploratory assessment of discriminative performance of candidate biomarkers and is suitable for preliminary evaluation of their discriminatory ability. However, the number of events remains limited for interaction testing and development of a multivariable prediction model; therefore, all related analyses should be considered exploratory.

Inclusion criteria were as follows: (1) diagnosis of childhood asthma according to the Global Initiative for Asthma (GINA) guidelines, defined by a history of recurrent wheezing, cough, or dyspnea with evidence of variable expiratory airflow limitation; (2) age between 3 and 15 years; and (3) complete laboratory test results at the time of blood sampling, including complete blood count, C-reactive protein (CRP), interleukin-6 (IL-6), immunoglobulin E (IgE), ferritin, and vitamin D.

Participants were divided into two groups according to their clinical status at the time of blood sampling:

Acute exacerbation group (n = 60): children presenting with acute asthma exacerbation symptoms (such as wheezing, dyspnea, or nocturnal awakening), with blood samples collected within 72 hours of symptom onset and no use of short-acting β-agonists (SABA) or systemic glucocorticoids within the preceding 6 hours.

Clinical remission group (n = 60): children with complete symptom control, a Childhood Asthma Control Test (c-ACT) score ≥ 20 (indicating well-controlled asthma), and clinical stability for ≥ 4 weeks.

All cases were recruited from routine clinical visits at the same institution, and blood samples were obtained in a single collection during clinical assessment. Samples from the exacerbation group were collected at the time of presentation, before any adjustment of treatment regimens; samples from the remission group were collected during routine evaluation in the stable state. Consequently, biomarker levels primarily reflect the current disease activity rather than changes induced by long-term treatment differences. The seasonal distribution of blood sampling was also compared between groups to evaluate potential confounding effects on vitamin D levels.

Exclusion criteria included: coexistence of other chronic respiratory diseases (such as cystic fibrosis or bronchopulmonary dysplasia); acute infection with body temperature > 38°C; primary or secondary immunodeficiency disorders; documented viral infection or allergen exposure within the preceding 4 weeks (based on history and allergen-specific IgE testing); exacerbations occurring during the seasonal peak period of asthma; or incomplete clinical data or missing key laboratory results.

The study was approved by the hospital ethics committee (approval number: LWOPN2024120602.0). Because the analysis utilized fully anonymized historical medical records, the requirement for informed consent was waived.

### 2.2 Laboratory measurements and methods

Blood samples were collected on the day of clinical assessment and, whenever possible, before any adjustment of the treatment regimen (e.g., addition or escalation of glucocorticoids or antibiotics) to reflect the biological characteristics associated with the child’s disease activity at that time.

All blood samples were sent for immediate testing. Complete blood count samples were analyzed within 30 minutes of collection. Samples requiring chemiluminescence immunoassay were centrifuged at 3000 rpm for 15 minutes to separate serum, which was then promptly loaded onto the analyzer.

Instruments and principles:

Complete blood count: Mindray BC-6800 Plus automated hematology analyzer (impedance method for red blood cell and platelet counts; laser flow cytometry for five-part white blood cell differential).

CRP: Mindray BC-6800 Plus CRP module (latex-enhanced immunoturbidimetric method).

IgE and IL-6: Roche Cobas e601 automated electrochemiluminescence immunoassay analyzer (electrochemiluminescence method).

Ferritin and vitamin D: Mindray CL-2000i automated chemiluminescence analyzer (chemiluminescence method).

Quality control: Before each run, high- and low-level quality control materials were used to verify instrument performance (IgE controls: Bio-Rad Liquichek™ levels 1 and 3; IL-6 controls: Roche PreciControl levels 1 and 2). Formal sample testing was performed only when quality control values fell within the target ± 2 standard deviations. Intra-assay precision (coefficient of variation, CV) for all measured analytes was < 5%.

### 2.3 Statistical analysis

Statistical analyses were performed using SPSS 25.0 and R 4.3.1 software. All tests were two-sided, and P < 0.05 was considered statistically significant. The distribution of continuous variables was assessed with the Shapiro–Wilk test. Normally distributed data are presented as mean ± standard deviation (mean ± SD) and compared using the independent-samples t-test; non-normally distributed data are expressed as median (interquartile range) and compared using the Mann–Whitney U test. Categorical variables are reported as frequencies and percentages, with between-group comparisons performed using the χ² test or Fisher’s exact test. Spearman correlation analysis was used to examine relationships among continuous variables, including vitamin D, ferritin, and eosinophil percentage. Acute asthma exacerbation (exacerbation = 1, stable = 0) served as the dependent variable. Univariate logistic regression was first conducted. Based on the study objectives and a P < 0.10 threshold, variables with variance inflation factor (VIF) < 5 were selected to assess multicollinearity; Ferritin, EOS%, and vitamin D were then entered into the multivariable logistic regression model, with additional adjustment for age and gender. To explore whether vitamin D modified the relationship between ferritin and exacerbation, an interaction term (Ferritin × VitD) was included. Stratified analysis was also performed according to 25(OH)D level (< 20 ng/mL vs. ≥ 20 ng/mL). Restricted cubic spline (RCS) models were constructed using the rms package in R to evaluate linear and non-linear associations of vitamin D and ferritin with exacerbation risk, with formal testing for non-linearity. Receiver operating characteristic (ROC) curves were generated to calculate the area under the curve (AUC) for individual markers and the combined model as measures of discriminative performance. Decision curve analysis (DCA) was used to assess the clinical net benefit of the model across different threshold probabilities.

## 3 Results

### 3.1 General characteristics

This study enrolled a total of 120 children with asthma, including 60 in the acute exacerbation group and 60 in the clinical remission group. There were no statistically significant differences between the two groups in age, gender distribution, or body mass index (BMI) (all P > 0.05). These results are presented in **Table 1**. These data indicate that the acute exacerbation and clinical remission groups were comparable in baseline demographic characteristics, thereby excluding potential confounding effects of age, gender, and obesity on subsequent analyses of inflammatory markers.

**Table 1:** Comparison of general characteristics between the two groups of children.

Group	Gender (Male, n/%)	Age (years)	BMI
Exacerbation Group	31/51.67	6.8 ± 3.5	19.27 ± 4.80
Stable Group	28/46.67	6.2 ± 3.1	19.34 ± 4.11
t/χ²	0.480	0.892	−0.081
P	0.489	0.375	0.936

To evaluate potential seasonal confounding of vitamin D levels, blood sampling dates were stratified according to meteorological seasons (spring: March–May; summer: June–August; autumn: September–November; winter: December–February). No significant difference in seasonal distribution was observed between the remission and exacerbation groups (χ² = 4.12, P = 0.255), suggesting that the timing of blood collection was comparable between groups and that differences in vitamin D levels were unlikely to result from uneven seasonal distribution. Results are shown in **Supplementary Table 1**.

### 3.2 Comparison of laboratory indices between the acute exacerbation and remission groups

Comparisons of complete blood count, inflammatory markers, immunological indices, and nutritional-metabolic indices between the two groups are presented in **Table 2**.

**Table 2:** Comparison of laboratory indicators between the stable group and the exacerbation group in children with asthma.

Indicator	Stable group (n = 60)	Exacerbation group (n = 60)	Test statistic (t/Z)	P value
Routine hematological indices				
Lymphocyte count (LY, ×10^9^/L)	3.12 ± 1.85	2.67 ± 1.92	t = 1.892	0.061
Eosinophil count (EOS, ×10^9^/L)	0.30 [0.12–0.45]	1.05 [0.68–1.50]	Z = 5.876	<0.001
Platelet count (PLT, ×10^9^/L)	328 ± 85	287 ± 92	t = 1.876	0.063
Neutrophil count (NEU, ×10^9^/L)	4.98 [3.21–6.75]	7.15 [5.22–10.33]	Z = 4.215	<0.001
Inflammatory biomarkers				
CRP (mg/L)	2.8 [1.6–4.3]	14.1 [7.5–21.8]	Z = 4.112	<0.001
IL-6 (pg/mL)	4.5 [2.3–7.1]	25.6 [12.4–38.7]	Z = 5.432	<0.001
Immune and metabolic indicators				
IgE (IU/mL)	185 [120–310]	735 [480–1120]	Z = 6.782	<0.001
Ferritin (ng/mL)	82 [45–125]	145 [85–210]	Z = 3.987	<0.001
Vitamin D (ng/mL)	24.3 ± 8.7	18.6 ± 7.2	t = 2.341	0.021
Inflammatory ratio indicators				
NLR (NEU/LY)	1.60 [1.05–2.15]	2.85 [1.92–3.90]	Z = 4.563	<0.001
EOS/NEU	0.06 [0.03–0.09]	0.14 [0.08–0.20]	Z = 5.112	<0.001
SII (PLT × NEU/LY)	540 [320–780]	1210 [850–1650]	Z = 5.221	<0.001

Normally distributed continuous variables are presented as mean ± standard deviation, and non-normally distributed variables are presented as median [interquartile range].

In routine hematological indices, the acute exacerbation group showed significantly higher eosinophil count (EOS) and neutrophil count (NEU) than the remission group (Z = 5.876, P < 0.001; Z = 4.215, P < 0.001), whereas lymphocyte count (LY) and platelet count (PLT) did not differ significantly (both P > 0.05).

Regarding inflammatory markers, C-reactive protein (CRP) and interleukin-6 (IL-6) levels were markedly elevated in the exacerbation group (Z = 4.112, P < 0.001; Z = 5.432, P < 0.001), indicating a more pronounced systemic inflammatory response during acute exacerbations.

In immunological and metabolic indices, immunoglobulin E (IgE) and ferritin (Ferritin) levels were significantly higher in the exacerbation group (Z = 6.782, P < 0.001; Z = 3.987, P < 0.001), while vitamin D levels were significantly lower (t = 2.341, P = 0.021).

Among cellular ratio indices, the neutrophil-to-lymphocyte ratio (NLR) and systemic immune-inflammation index (SII = PLT × NEU/LY) were both significantly elevated in the exacerbation group (Z = 4.563, P < 0.001; Z = 5.221, P < 0.001), as was the eosinophil-to-neutrophil ratio (Eo/Neu) (Z = 5.112, P < 0.001). These findings indicate that children with acute asthma exacerbations exhibit systemic inflammation characterized by neutrophil and eosinophil activation, accompanied by upregulation of acute-phase reactants and a decline in vitamin D levels.

### 3.3 Correlation analysis of indices

To further explore the interrelationships among indices associated with acute asthma exacerbations in children, correlation analyses were performed on inflammatory, immunological, and nutritional markers (key correlations are shown in **Table 3**; all correlations are presented in **Supplementary Table 2**).

**Table 3:** Spearman correlation analysis among key inflammatory and metabolic indicators.

Variable 1	Variable 2	r	P value
IL-6	CRP	0.68	<0.001
EOS%	Ferritin	0.30	<0.001
Ferritin	VitD	-0.31	<0.001
EOS	VitD	-0.31	0.01
Ferritin	EOS	0.29	<0.001

Ferritin was positively correlated with eosinophilic inflammatory indicators and negatively correlated with vitamin D, suggesting a potential link between inflammatory activity and metabolic status.

Results showed that 25(OH)D levels were significantly negatively correlated with ferritin (r = −0.31, P < 0.001) and showed a weak negative trend with eosinophil percentage (r = −0.17, P = 0.06). Ferritin was positively correlated with eosinophil percentage (r = 0.29, P < 0.001) and with IgE (r = 0.23, P = 0.01).

Among inflammatory markers, the strongest correlation was observed between CRP and IL-6 (r = 0.68, P < 0.001). CRP was negatively correlated with lymphocyte count (r = −0.25, P = 0.01) and moderately positively correlated with SII and NLR (both P < 0.05). In addition, eosinophil count and EOS/LY showed weak correlations with IL-6 (P < 0.05) but were not significantly correlated with vitamin D. These results suggest that lower vitamin D levels are associated to some extent with higher ferritin and eosinophil activation, with ferritin potentially occupying a central position at the intersection of inflammation and immune activation.

### 3.4 Logistic regression analysis of acute exacerbation risk

With acute asthma exacerbation as the dependent variable (exacerbation = 1, stable = 0), univariate logistic regression was performed for each clinical index (**Table 4**). Multiple inflammatory and immunological indices were significantly associated with acute exacerbation. Eosinophil count (EOS), eosinophil percentage (EOS%), and the EOS/NEU ratio all showed significant positive associations (all P < 0.001) with relatively large effect sizes, indicating a central role of eosinophilic inflammation in acute exacerbations. Ferritin levels were also significantly positively associated with exacerbation (OR = 1.093, 95% CI: 1.060–1.128, P < 0.001), suggesting that iron metabolism-related inflammatory status may contribute to exacerbation risk.

**Table 4:** Univariate logistic regression analysis for acute asthma exacerbation in children.

Variable	β	OR	95% CI (OR)	Z value	P-value
EOS (×10^9^/L)	3.167	23.72	7.66–73.47	5.49	<0.001
Eosinophil percentage (EOS%)	0.359	1.432	1.244–1.649	4.99	<0.001
EOS/NEU	6.035	417.65	18.60–9378.84	3.80	<0.001
Ferritin (ng/mL)	0.089	1.093	1.060–1.128	5.62	<0.001
Vitamin D (ng/mL)	−0.112	0.894	0.845–0.946	−3.87	<0.001
IgE (IU/mL)	0.0014	1.0014	1.0003–1.0026	2.43	0.015
ln(IL-6)	0.236	1.266	0.989–1.619	1.88	0.061
NEU (×10^9^/L)	−0.065	0.937	0.511–1.324	−0.21	0.833
CRP (mg/L)	−0.026	0.974	0.834–1.137	−0.33	0.738
NLR (NEU/LY)	−0.015	0.985	0.897–1.081	−0.32	0.747
SII (PLT × NEU/LY)	−0.0004	1.000	0.948–1.053	−0.02	0.988
Age (years)	0.022	1.023	0.907–1.153	0.37	0.714
Gender (male = 1)	−0.071	0.931	0.445–1.951	−0.19	0.850

Vitamin D showed a significant negative association in the univariate analysis (OR = 0.894, 95% CI: 0.845–0.946, P < 0.001), suggesting a potential protective effect. IgE displayed a modest positive association (OR = 1.0014, P = 0.015). In contrast, ln(IL-6) was only marginally significant (P = 0.061), while NEU, CRP, NLR, SII, and demographic variables (age and gender) showed no significant associations (all P > 0.05).

Given the large number of variables in the univariate analysis and the limited number of events (which could lead to model overfitting), a simplified modeling strategy was adopted for multivariable analysis. Only the core variables of clinical relevance (Ferritin, EOS%, and vitamin D) together with basic demographic factors (age and gender) were included (**Table 5**). After adjustment for age and gender, Ferritin (OR = 1.118, 95% CI: 1.066–1.173, P < 0.001) and EOS% (OR = 1.831, 95% CI: 1.322–2.536, P < 0.001) remained independent risk factors for acute asthma exacerbation in children.

**Table 5:** Multivariable logistic regression analysis of acute asthma exacerbation in children.

Variable	β	OR	95% CI for OR	P value
Ferritin (per 1 ng/mL)	0.112	1.118	1.066–1.173	<0.001
Eosinophil percentage (EOS%, per 1%)	0.605	1.831	1.322–2.536	<0.001
Vitamin D (per 1 ng/mL)	-0.085	0.918	0.833–1.012	0.086
Age (years)	-0.043	0.958	0.736–1.246	0.747
Gender (male = 1)	0.235	1.265	0.259–6.189	0.772

Vitamin D retained a negative directional trend in the multivariable model (OR = 0.918, 95% CI: 0.833–1.012) but did not reach statistical significance (P = 0.086), suggesting that its effect may be influenced by other inflammatory and metabolic factors and is more likely to act as a modulating factor in the exacerbation process. Age and gender showed no significant effects in the model.

Overall, univariate analysis indicated that multiple inflammatory and immunological indices were associated with acute exacerbation, while the multivariable model further identified eosinophilic inflammation and iron metabolism-related indices as the primary independent contributors, with vitamin D showing a potential protective trend but limited independent effect.

### 3.5 Interaction and stratified analysis of vitamin D and ferritin

To explore the potential modulating role of vitamin D in the relationship between inflammation-metabolic indices and acute asthma exacerbations, interaction terms (Ferritin × VitD and EOS% × VitD) were constructed on the basis of the simplified multivariable logistic regression model (including Ferritin, EOS%, vitamin D, age, and gender). All continuous variables were mean-centered prior to analysis.

Neither interaction term reached statistical significance: Ferritin × VitD (β = −0.0015, OR = 0.998, 95% CI: 0.993–1.004, P = 0.555) and EOS% × VitD (β = 0.0095, OR = 1.010, 95% CI: 0.974–1.046, P = 0.604). Model fit was not significantly improved compared with the base model (likelihood ratio χ² tests, both P > 0.05).

Given the limited sample size and number of events in this study, the above interaction analyses are considered exploratory only. They indicate that no significant modulating effect of vitamin D on the relationships between ferritin or eosinophil percentage and acute exacerbation was observed in the current sample; however, a potential interaction cannot be excluded and requires further verification in larger studies (**Table 6**).

**Table 6:** Interaction analysis (Interaction Models).

Variable	Model A (Main Effects) β (OR, 95% CI)	Model B (Ferritin × VitD)	Model C (EOS% × VitD)
Ferritin (per 1 ng/mL)	0.1236, OR = 1.132 (1.071–1.183), P < 0.001	0.118, OR = 1.125 (1.062–1.190), P < 0.001	0.119, OR = 1.127 (1.064–1.193), P < 0.001
EOS% (per 1%)	0.7003, OR = 2.014 (1.338–2.698), P < 0.001	0.662, OR = 1.939 (1.296–2.913), P = 0.001	0.691, OR = 1.996 (1.340–3.007), P < 0.001
VitD (per 1 ng/mL)	−0.0795, OR = 0.924 (0.837–1.019), P = 0.114	−0.052, OR = 0.949 (0.870–1.035), P = 0.237	−0.050, OR = 0.951 (0.873–1.035), P = 0.242
Ferritin × VitD	—	−0.0015, OR = 0.998 (0.993–1.004), P = 0.555	—
EOS% × VitD	—	—	0.0095, OR = 1.010 (0.974–1.046), P = 0.604
LRT vs Model A	—	χ² = 0.35, P = 0.558	χ² = 0.26, P = 0.605

Stratified analysis was further performed according to 25(OH)D level (< 20 ng/mL vs. ≥ 20 ng/mL), dividing the children into a low vitamin D group (VitD < 20 ng/mL, n = 49) and a normal group (VitD ≥ 20 ng/mL, n = 71). Results showed that ferritin levels were higher in the low vitamin D group [104.11 (83.88–129.86) vs. 86.81 (69.86–113.92) ng/mL, P = 0.016]. IL-6, eosinophil count, and EOS% did not differ significantly between the two groups (all P > 0.05) but showed a trend toward elevation in the vitamin D-deficient group.

It should be noted that the above stratified analysis was descriptive only and did not include multivariable adjustment; therefore, the results should be interpreted with caution (**Table 7**).

**Table 7:** Comparison of key biomarkers stratified by vitamin D levels.

Variable	VitD ≥20 ng/mL	VitD <20 ng/mL	P-value
Ferritin (ng/mL)	86.81 (69.86–113.92)	104.11 (83.88–129.86)	0.016
IL-6	3.20 (1.52–10.83)	3.75 (1.58–16.17)	0.250
EOS	0.39 (0.14–0.95)	0.51 (0.21–1.16)	0.123
EOS%	4.71 (1.90–6.85)	6.00 (2.70–8.11)	0.232

### 3.6 ROC curve, RCS dose-response, and DCA analyses

#### 3.6.1 ROC curve analysis

**Figure 1:**
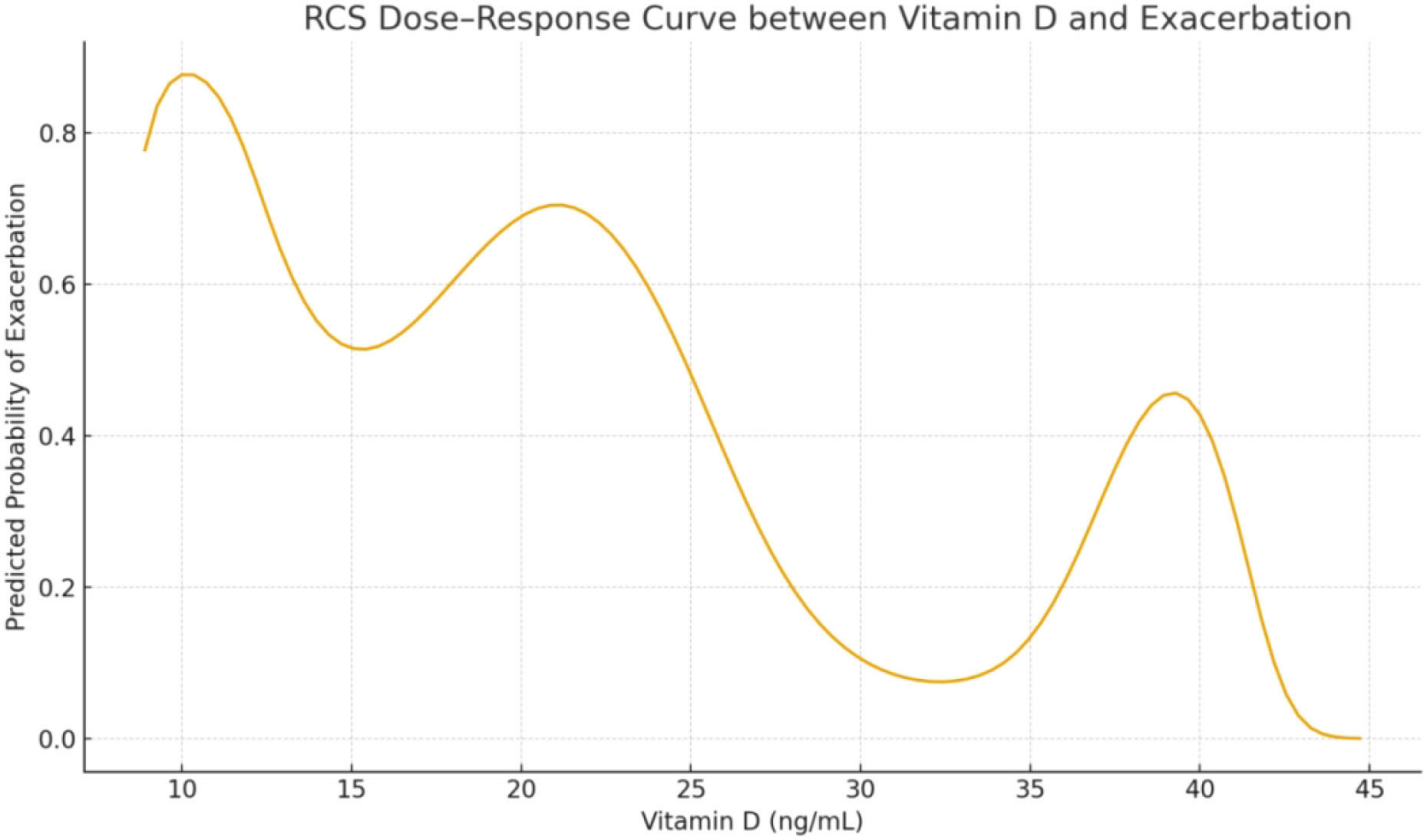
RCS dose-response curve (Vitamin D vs. acute asthma exacerbation).

**Figure 2:**
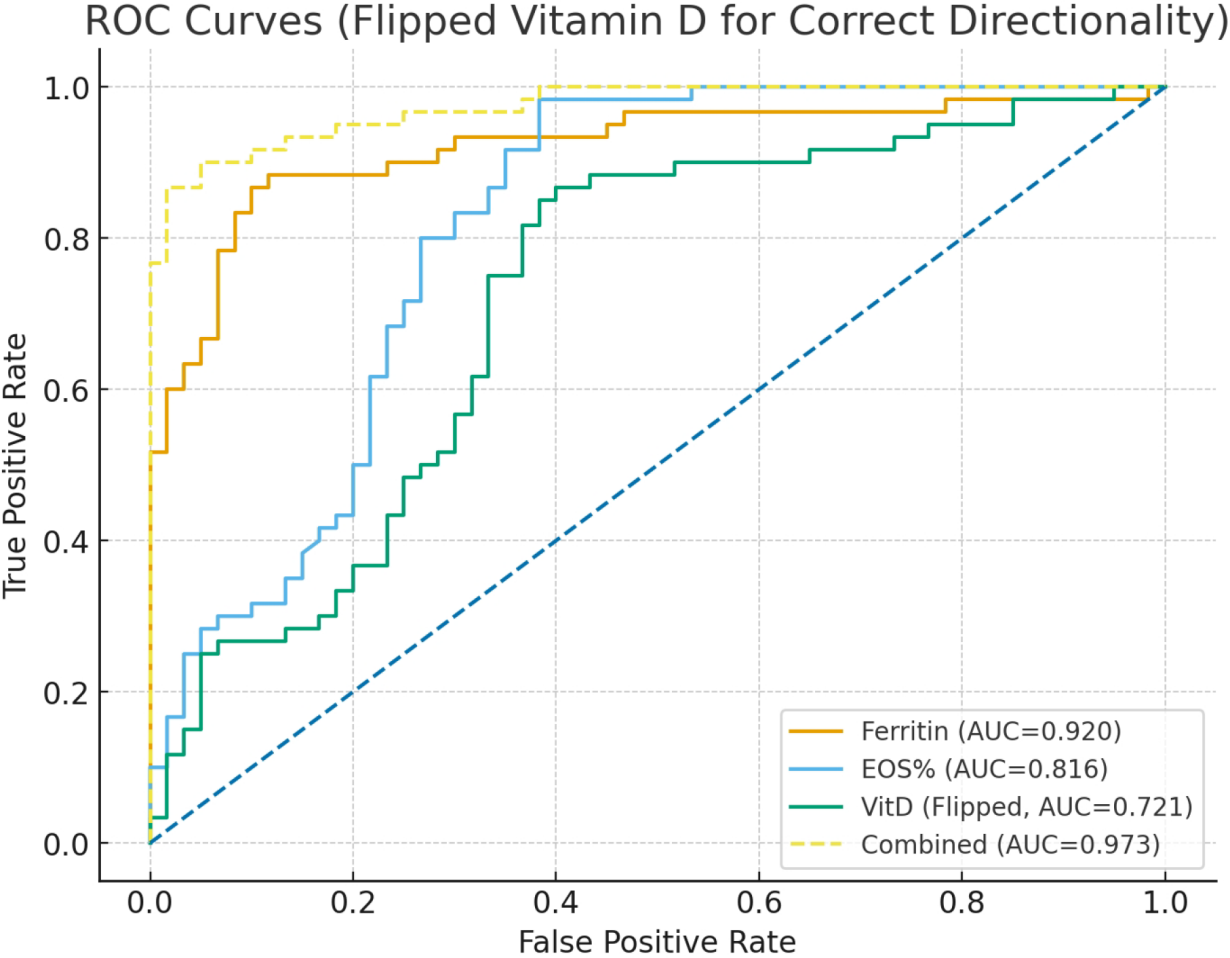
ROC curves of ferritin, eosinophil percentage, vitamin D, and their combined model for predicting acute exacerbations of childhood asthma.

Receiver operating characteristic (ROC) curves were constructed to evaluate the discriminative ability of different indices for acute asthma exacerbation in children. Single-marker analysis showed that ferritin (Ferritin) had the highest discriminative ability for acute exacerbation (AUC = 0.920), followed by eosinophil percentage (EOS%; AUC = 0.816), while vitamin D (VitD) had relatively lower discriminative performance (AUC = 0.721) (see **Figure 1**). When Ferritin, EOS%, VitD, age, and gender were jointly included in the multivariable logistic model, the AUC increased substantially to 0.974, outperforming any single marker and indicating superior discriminative capacity of the combined model (**Table 8**, **Figure 2**). It should be noted that, owing to the limited sample size and number of events, the discriminative performance of this model may exhibit some optimism bias; results should therefore be interpreted in conjunction with internal validation findings.

**Table 8:** ROC curve analysis of ferritin, eosinophil percentage, vitamin D, and their combined model for predicting acute asthma exacerbation.

Variable	AUC	AUC 95% CI lower	AUC 95% CI upper	Optimal Cut-off	Sensitivity	Specificity
Ferritin (ng/mL)	0.92	0.864	0.966	95.75	0.867	0.900
EOS% (per 1%)	0.816	0.732	0.887	3.15	0.983	0.617
Vitamin D (reversed)	0.721	0.628	0.814	24.5	0.867	0.600
Combined (Ferritin + EOS% + Vitamin D)	0.973	0.952	0.994	0.747	0.867	0.983

#### 3.6.2 Restricted cubic spline dose–response analysis

**Figure 3:**
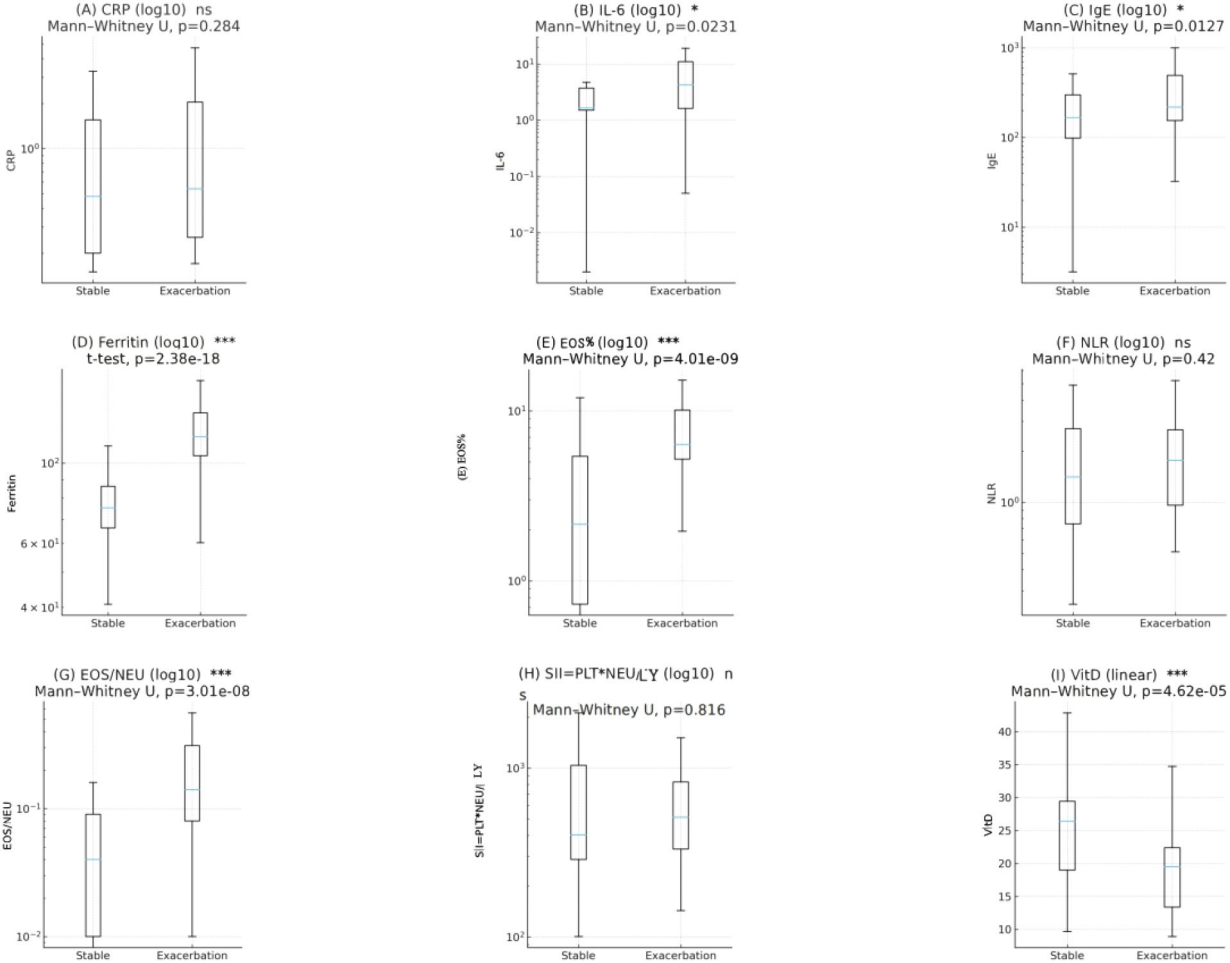
Comparison of inflammatory and metabolic biomarkers between the stable and acute exacerbation groups. **(A)** CRP, **(B)** IL-6, **(C)** IgE, **(D)** ferritin, **(E)** EOS%, **(F)** NLR, **(G)** EOS/NEU, **(H)** SII, and **(I)** vitamin D. Box plots show the distribution of each biomarker in the two groups. P values are indicated in each panel. *, P < 0.05; ***, P < 0.001.

Three-knot RCS models were used to analyze the relationships between continuous variables and exacerbation risk. After multivariable adjustment, vitamin D levels showed a linear inverse trend with exacerbation risk (non-linearity test P = 0.341; no significant non-linear effect was detected; see **Figure 3**). Ferritin exhibited a linear positive association with exacerbation risk (non-linearity test P = 0.105; no obvious non-linear relationship; see **Figure 3**). These results indicate that both decreasing vitamin D and increasing ferritin are linearly associated with increased risk of acute asthma exacerbation (results shown in **Figure 1**). Given the limited sample size, the RCS analysis in this study was primarily exploratory for potential dose–response relationships and requires further validation in larger cohorts.

#### 3.6.3 Decision curve analysis

**Figure 4:**
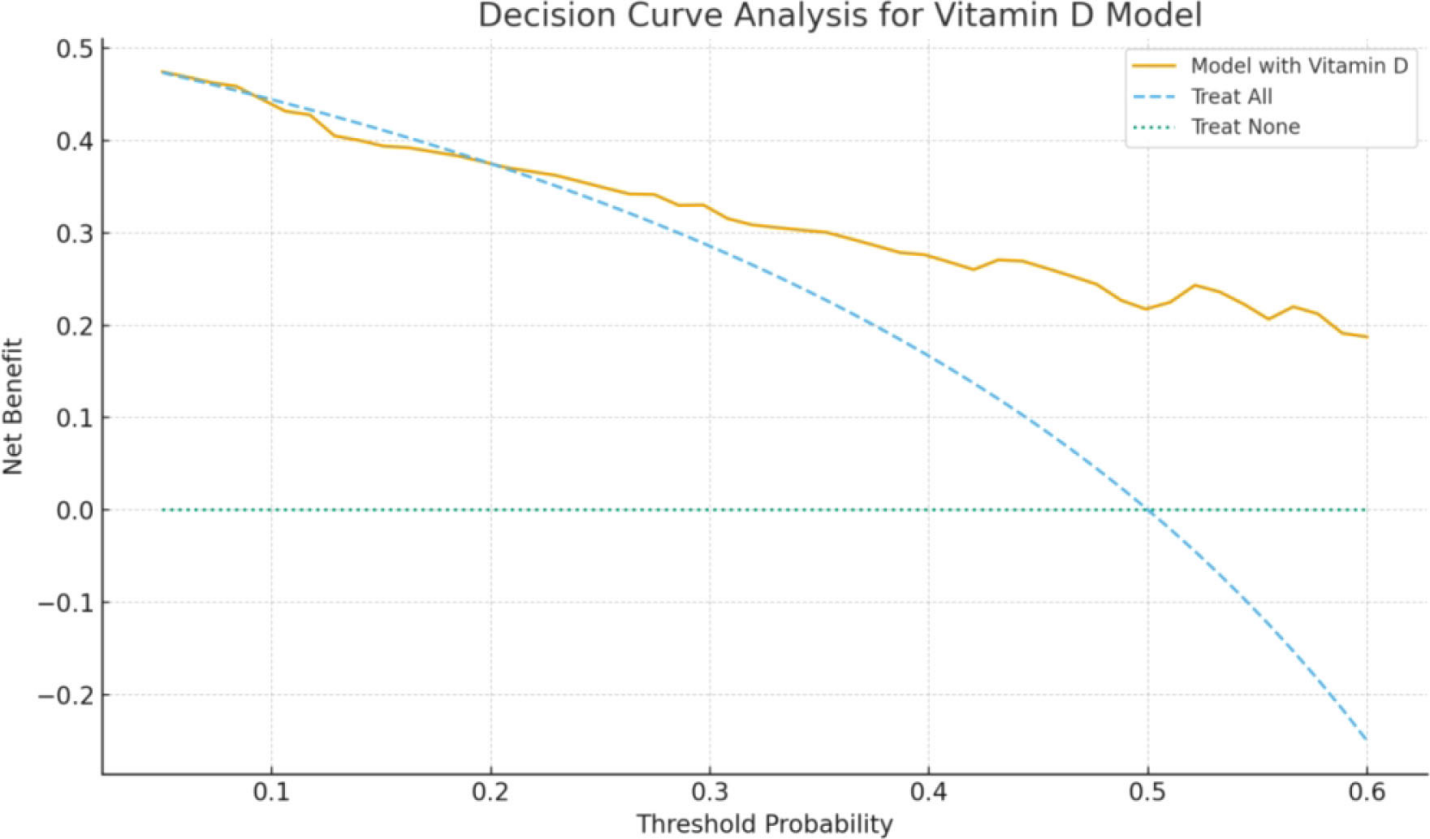
Decision curve analysis (DCA).

To assess the clinical utility of the model, decision curve analysis was performed on the combined Ferritin + EOS% + VitD model. The results demonstrated that, across threshold probabilities of 0.20–0.70, the net benefit of the model exceeded that of both the “treat-all” and “treat-none” strategies (see **Figure 4**), suggesting potential clinical value for decision-making. However, considering the degree of overfitting risk in the model, the observed clinical benefit still requires further validation in independent external datasets.

### 3.7 Internal model validation

To evaluate the robustness and predictive reliability of the multivariable logistic regression model, internal validation was performed using the bootstrap method (500 resamples) (**Table 9**). The apparent AUC was 0.978, with a bootstrap-corrected AUC of 0.962. The Brier score was corrected from 0.062 to 0.088. The model demonstrated good discriminative ability in the current study sample; however, bootstrap internal validation indicated substantial optimism bias and overfitting risk (calibration slope = 0.543). Therefore, its predictive performance and clinical applicability require further validation in larger samples and independent external cohorts.

**Table 9:** Bootstrap internal validation results (B = 500).

Metric	Apparent	Average optimism (B = 500)	Optimism-corrected
AUC	0.978	0.016	0.962
Brier score	0.062	-0.025	0.088
Calibration intercept	0.0	-0.032	0.032
Calibration slope	1.0	0.457	0.543

AUC refers to the area under the receiver operating characteristic (ROC) curve; the Brier score reflects the deviation between predicted probabilities and actual outcomes; the calibration intercept and slope are used to assess the goodness of fit of the model.

Taken together, the discrimination and calibration results indicate that the simplified model based on Ferritin, EOS%, VitD, and basic demographic variables possesses certain predictive capacity, but its stability and generalizability still require further evaluation in larger samples and external cohorts.

## 4 Discussion

This study investigated the immune-nutritional interactions among vitamin D, ferritin, and eosinophilic inflammation in childhood asthma and constructed a predictive model for acute exacerbation risk. The results showed that, after controlling for potential confounding by age and gender and confirming no significant difference in seasonal distribution of blood sampling between groups, serum ferritin (Ferritin) and eosinophil percentage (EOS%) were independent risk factors for acute exacerbations of childhood asthma. Vitamin D exhibited a negative trend with exacerbation risk but did not reach statistical significance after multivariable adjustment, suggesting that it is more likely to function as an immune-metabolic modulator rather than an independent predictor. Restricted cubic spline (RCS) analysis indicated approximately linear relationships between both Ferritin and VitD and exacerbation risk: higher Ferritin and lower VitD were associated with elevated risk, although these dose–response relationships should be interpreted cautiously. The combined model integrating Ferritin, EOS%, and VitD demonstrated excellent discriminative performance (AUC = 0.973). However, given the limited sample size and number of events, this result may be subject to optimism bias. The model achieved the greatest net benefit in decision curve analysis, indicating potential clinical utility, but further validation is still required. Moreover, because this was a single-center retrospective study and internal validation revealed a degree of overfitting, the generalizability of the model requires confirmation in larger samples and multicenter cohorts.

The finding of elevated Ferritin in association with acute asthma exacerbations is consistent with recent research on the role of iron metabolism dysregulation in airway inflammation. Ferritin is not only an intracellular iron-storage protein but also an acute-phase reactant induced by IL-6 and oxidative stress (19, 20). In the inflammatory state of asthma, cytokines can activate macrophages and airway epithelial cells, promoting Ferritin synthesis and resulting in iron retention. Excess intracellular iron can catalyze the generation of reactive oxygen species, exacerbating oxidative damage and airway epithelial injury (21). Several pediatric studies have also reported significantly higher Ferritin and hepcidin levels during asthma exacerbations compared with the remission phase (22, 23). The present study further confirmed that, even after adjustment for age, gender, and core inflammation-related indices in the multivariable model, Ferritin remained an independent predictor of acute exacerbation. This suggests that Ferritin possesses dual metabolic and inflammatory biological characteristics and supports the possibility that iron homeostasis dysregulation is linked to acute exacerbations of childhood asthma. Nevertheless, given the single-center retrospective design and the failure to collect data on long-term controller medication use and individual sunlight exposure as potential confounders, the current results more strongly support Ferritin as a robust biomarker associated with acute exacerbation rather than establishing an independent pathophysiological role, which requires further validation through prospective studies and mechanistic experiments.

Eosinophilic inflammation remains a core feature of type 2-high (T2-high) asthma. This study found that each 1% increase in EOS% was associated with a significant increase in exacerbation risk (OR ≈ 2.1), consistent with previous multicenter cohort findings that elevated peripheral blood eosinophils are closely related to exacerbation frequency and glucocorticoid responsiveness (24, 25). Eosinophils can release cytotoxic products such as eosinophil cationic protein (ECP) and leukotrienes, directly damaging airway epithelium and sustaining inflammation. Notably, the correlation between Ferritin and EOS% in this study was relatively weak, suggesting that the two markers represent distinct dimensions of metabolic versus cellular inflammation. Incorporating both Ferritin and EOS% into the multivariable model improved discriminative performance, indicating that integration of multidimensional inflammatory indices may more comprehensively reflect the risk profile of acute asthma exacerbations; however, this improvement may still be influenced by sample size and model complexity and should be interpreted cautiously. These results indirectly support the heterogeneous nature of airway inflammation in childhood asthma, although the underlying mechanisms require further investigation.

The role of vitamin D in asthma remains controversial. In the present study, 25(OH)D levels were significantly lower in the exacerbation group than in the remission group, but the association weakened in the multivariable model. Possible explanations include: first, vitamin D deficiency may represent a long-term susceptibility factor rather than an acute trigger, and its effect may be masked by more direct inflammatory indices such as Ferritin or EOS%; second, seasonal and sunlight exposure differences are difficult to fully control in retrospective studies and may introduce residual confounding; in addition, this study did not systematically collect data on long-term use and adherence to inhaled corticosteroids (ICS) or leukotriene receptor antagonists (LTRA), factors that could also influence the estimated association between vitamin D and exacerbation risk; third, the study used a 25(OH)D threshold of < 20 ng/mL to define deficiency, based primarily on international endocrine guidelines, but this cutoff may not fully capture “functional insufficiency” at the airway immune level, and differences in vitamin D-binding protein genotypes across populations may further affect biological effects (26). Existing systematic reviews have reached inconsistent conclusions regarding the benefits of vitamin D supplementation in childhood asthma, indicating that its clinical impact may be modulated by multiple factors including baseline vitamin D status, study population characteristics, and study design. Furthermore, continuous-variable and RCS analyses in this study revealed a linear inverse trend between vitamin D and exacerbation risk, yet this association did not reach statistical significance after multivariable adjustment, suggesting that the effect may be relatively modest or that statistical power was limited by sample size. Based on these findings, vitamin D cannot be regarded as an independent predictor of acute exacerbations in childhood asthma. Although previous studies have suggested that vitamin D may participate in Th2 inflammation regulation, immune tolerance maintenance, and airway remodeling (27, 28), these mechanisms were not directly verified in the present study. Consequently, the role of vitamin D in childhood asthma in this investigation is more consistent with an immune-nutritional background factor associated with disease state rather than a clear independent trigger or protective factor for acute exacerbations.

No statistically significant interaction was observed between vitamin D and either Ferritin or EOS%. However, given the current sample size and limited power for interaction testing, a potential biological link cannot be ruled out. Experimental studies have shown that vitamin D can suppress IL-6-mediated hepcidin expression, thereby promoting iron export and reducing intracellular Ferritin accumulation (29). In states of vitamin D deficiency, this regulatory mechanism is impaired, potentially leading to iron retention, enhanced oxidative stress, and aggravation of the airway inflammatory microenvironment. It should be noted that the interaction analyses in this study were based on product terms of continuous variables, and statistical power may have been constrained by sample size. In the stratified analysis, the association between Ferritin and exacerbation risk appeared stronger in the low vitamin D group, but between-group differences did not reach statistical significance; therefore, these results are exploratory only and insufficient to support a definitive effect-modifying role of vitamin D. Future studies should incorporate measurements of hepcidin, vitamin D receptor (VDR) expression, and oxidative stress markers to further validate the regulatory role of vitamin D in iron-driven inflammation and use prospective designs to clarify whether vitamin D exerts an effect-modifying function.

The model constructed in this study exhibited high discriminative power, with the Ferritin + EOS% + VitD combined model yielding an AUC of 0.973, indicating good separation in the current sample. However, the bootstrap-corrected calibration slope of 0.543 after internal validation suggests persistent overfitting risk; therefore, predictive performance should be interpreted cautiously. Decision curve analysis showed that the combined model provided the greatest net benefit across clinical threshold probabilities of 0.2–0.7, indicating potential clinical value in this sample, although this finding still requires validation in independent external populations. Ferritin and EOS% measurements are simple and inexpensive, and vitamin D testing is routine; thus, the model is feasible from the standpoint of marker acquisition. Nevertheless, before widespread application, its stability, generalizability, and suitability across different clinical scenarios must be further evaluated. If incorporated into routine monitoring, such indices could assist in identifying high-risk children and provide references for optimizing anti-inflammatory therapy and vitamin D-related intervention studies, but the actual effectiveness of any intervention requires prospective validation.

RCS dose–response analysis further revealed a linear positive association between Ferritin and exacerbation risk, suggesting that even moderate elevations may increase risk rather than an effect limited to high levels. Vitamin D showed a monotonic inverse association with exacerbation risk, indicating that higher 25(OH)D levels are directionally linked to lower risk; however, because this association did not reach statistical significance after multivariable adjustment, it should not be interpreted as an independent protective effect. No obvious non-linear inflection points were identified, supporting the reasonableness of using linear terms to model the relationships between these indices and exacerbation risk in the present sample. Overall, the RCS and conventional regression results were consistent in direction of association, but given the limited sample size and overfitting risk, these findings should be interpreted with caution. Clinically, monitoring serum vitamin D and ferritin levels may help supplement immune-metabolic assessment of inflammatory status and acute exacerbation risk in children with asthma, although clinical benefit still requires further verification. Although the efficacy of vitamin D supplementation has varied across clinical trials, the present study suggests that children with concurrent low vitamin D and iron metabolism abnormalities may represent a potentially high-risk subgroup worthy of further investigation. Future prospective studies should examine whether vitamin D intervention can modulate the IL-6–hepcidin–Ferritin axis and improve asthma control and clarify its potential role across different inflammatory phenotypes.

It should be noted that this study did not systematically record the dosage or adherence to long-term controller medications (such as inhaled corticosteroids or leukotriene receptor antagonists), which may constitute potential confounding factors. Previous studies have shown that ICS therapy can reduce peripheral eosinophil levels; therefore, an effect on indices such as EOS% cannot be entirely excluded. However, blood sampling in this study was performed at initial presentation for acute exacerbations or during stable-phase evaluations, and the exacerbation group excluded patients who had received short-term systemic glucocorticoids. Consequently, the measured indices are more likely to reflect current disease activity rather than immediate drug effects. Nevertheless, cumulative effects from long-term controller medication exposure could not be fully distinguished. Importantly, as an IL-6-related acute-phase reactant, ferritin levels are primarily influenced by inflammation and iron homeostasis and are less directly affected by routine ICS therapy. In the multivariable model, Ferritin retained statistical independence after adjustment for inflammatory indices and demographic variables, suggesting that its relationship with exacerbation is not entirely dependent on the treatment-related differences already included in the model. However, given the absence of long-term treatment information, residual confounding cannot be ruled out. Despite these considerations, residual confounding from long-term medication differences cannot be completely excluded, and future prospective studies should systematically incorporate treatment variables for validation.

This study also has several limitations. First, it was a single-center retrospective design with a limited sample size; statistical power was therefore insufficient for interaction testing and evaluation of prediction model stability, and causal inference requires prospective confirmation. Second, although seasonal stratification of blood sampling was performed, individual sunlight exposure, dietary intake, and long-term controller medication use could not be fully quantified or controlled, potentially leaving residual confounding. Third, hepcidin, VDR, and oxidative stress markers were not measured, limiting mechanistic inferences. Fourth, despite bootstrap internal validation, the model still showed overfitting risk and therefore requires external validation in multicenter cohorts of different ethnicities. Finally, vitamin D supplementation was not standardized among patients; future interventional studies are needed to evaluate whether vitamin D correction can influence iron metabolism-related indices and asthma exacerbation risk.

Despite these limitations, the study possesses certain novelty. Few studies have integrated nutritional, inflammatory, and metabolic indices (vitamin D, Ferritin, and eosinophils) within a unified predictive framework for childhood asthma. The application of RCS, interaction terms, and DCA provided multi-level exploration of the relationships between these indices and acute exacerbation risk. Ferritin was identified as one of the independent candidate markers associated with exacerbation, highlighting the potential importance of metabolic inflammation and iron homeostasis in asthma pathophysiology. Clinically, the combined model demonstrated risk-stratification potential in this sample and may serve as a reference for early identification of high-risk children and subsequent individualized intervention studies, although its actual clinical utility requires further validation.

## 5 Conclusion

In summary, elevated Ferritin and EOS% are independent risk factors for acute exacerbations of childhood asthma. Vitamin D showed an inverse directional association in univariate and dose–response analyses but was not an independent predictor after multivariable adjustment. Correlations exist among vitamin D levels, ferritin, and eosinophilic inflammation indices, suggesting that metabolic status and inflammatory activity may jointly contribute to acute exacerbations of childhood asthma. Incorporating these indices into a predictive model improved risk-assessment discrimination in the current sample, but its stability and generalizability still require further testing. Future prospective studies and mechanistic experiments are needed to validate the regulatory role of vitamin D in iron metabolism pathways and to clarify its applicability across different inflammatory phenotypes and clinical contexts, with the ultimate goal of providing more precise theoretical support for the prevention and management of childhood asthma.

## Data Availability

The de-identified raw data supporting the conclusions of this article will be made available by the authors upon reasonable request, subject to institutional approval and ethical requirements, without undue reservation.

## Supplementary material

Supplementary Table 1 Seasonal distribution of blood sampling between stable and exacerbation groups.

Supplementary Table 2 Correlation analysis among different indicators in children with asthma CRP, C-reactive protein; EOS, eosinophil count; Eo/LY, eosinophil-to-lymphocyte ratio; IL-6, interleukin-6; IgE, immunoglobulin E; LY, lymphocyte count; NLR, neutrophil-to-lymphocyte ratio; SII, systemic immune-inflammation index; EOS%, eosinophil percentage; VitD, vitamin D. Correlation coefficients were calculated using Spearman’s rank correlation analysis. *P < 0.05, **P < 0.01, ***P < 0.001.
