# Supplementary material for: Integrative analysis of vitamin D, ferritin, and eosinophilic inflammation in predicting acute exacerbations of childhood asthma
Source: Front Immunol. 2026 Apr 20;17:1746377. doi: 10.3389/fimmu.2026.1746377 (PMC13135990; doi:10.3389/fimmu.2026.1746377)
Supplement: Supplementary Table 1 — Seasonal distribution of blood sampling between stable and exacerbation groups. [file Table1.docx]

Supplementary Table 1 Seasonal distribution of blood sampling between stable and exacerbation groups

| Season | Stable (n=60) | Exacerbation (n=60) |
| --- | --- | --- |
| Spring | 20 | 12 |
| Summer | 12 | 13 |
| Autumn | 18 | 27 |
| Winter | 10 | 8 |
| Overall comparison: χ² test, χ² = 4.12, P = 0.255 | | |
