# Supplementary material for: Integrative analysis of vitamin D, ferritin, and eosinophilic inflammation in predicting acute exacerbations of childhood asthma
Source: Front Immunol. 2026 Apr 20;17:1746377. doi: 10.3389/fimmu.2026.1746377 (PMC13135990; doi:10.3389/fimmu.2026.1746377)
Supplement: Supplementary Table 2 — Correlation analysis among different indicators in children with asthma CRP, C-reactive protein; EOS, eosinophil count; Eo/LY, eosinophil-to-lymphocyte ratio; IL-6, interleukin-6; IgE, immunoglobulin E; LY, lymphocyte count; NLR, neutrophil-to-lymphocyte ratio; SII, systemic immune-inflammation index; EOS%, eosinophil percentage; VitD, vitamin D. Correlation coefficients were calculated using Spearman’s rank correlation analysis. *P < 0.05, **P < 0.01, ***P < 0.001. [file Table2.docx]

Supplementary Table 2 Correlation analysis among different indicators in children with asthma

| **Biomarker** |  | **CRP (mg/L)** | **EOS (×10^9/L)** | **Eo/LY** | **IL-6** | **IgE** | **LY (×10^9/L)** | **NLR** | **SII** | **EOS%** | **VitD** | **Ferritin** |
| --- | --- | --- | --- | --- | --- | --- | --- | --- | --- | --- | --- | --- |
| **CRP (mg/L)** | r | – | -0.02 | 0.10 | 0.68*** | 0.17 | -0.25* | 0.25* | 0.21* | 0.01 | 0.01 | -0.04 |
|  | P | – | 0.85 | 0.28 | <0.001 | 0.06 | 0.01 | 0.01 | 0.02 | 0.94 | 0.96 | 0.67 |
| **EOS (×10^9/L)** | r | -0.02 | – | 0.72*** | 0.00 | 0.11 | 0.05 | -0.11 | -0.07 | 0.54*** | -0.31** | 0.29** |
|  | P | 0.85 | – | <0.001 | 0.97 | 0.24 | 0.58 | 0.22 | 0.44 | <0.001 | 0.01 | 0.00 |
| **Eo/LY** | r | 0.10 | 0.72*** | – | 0.19* | 0.15 | -0.38*** | 0.28** | 0.32*** | 0.37*** | -0.17 | 0.29** |
|  | P | 0.28 | <0.001 | – | 0.04 | 0.10 | <0.001 | 0.00 | 0.00 | <0.001 | 0.06 | 0.00 |
| **IL-6** | r | 0.68*** | 0.00 | 0.19* | – | 0.17 | -0.11 | 0.17 | 0.10 | 0.07 | 0.02 | -0.02 |
|  | P | <0.001 | 0.97 | 0.04 | – | 0.06 | 0.25 | 0.06 | 0.28 | 0.46 | 0.80 | 0.83 |
| **IgE** | r | 0.17 | 0.11 | 0.15 | 0.17 | – | -0.16 | 0.09 | 0.09 | 0.39*** | -0.10 | 0.23* |
|  | P | 0.06 | 0.24 | 0.10 | 0.06 | – | 0.08 | 0.35 | 0.31 | <0.001 | 0.29 | 0.01 |
| **LY (×10^9/L)** | r | -0.25* | 0.05 | -0.38*** | -0.11 | -0.16 | – | -0.55*** | -0.51*** | -0.01 | -0.07 | -0.08 |
|  | P | 0.01 | 0.58 | <0.001 | 0.25 | 0.08 | – | <0.001 | <0.001 | 0.95 | 0.44 | 0.38 |
| **NLR** | r | 0.25* | -0.11 | 0.28** | 0.17 | 0.09 | -0.55*** | – | 0.97*** | -0.19* | 0.08 | 0.01 |
|  | P | 0.01 | 0.22 | 0.00 | 0.06 | 0.35 | <0.001 | – | <0.001 | 0.04 | 0.40 | 0.94 |
| **SII** | r | 0.21* | -0.07 | 0.32*** | 0.10 | 0.09 | -0.51*** | 0.97*** | – | -0.19* | 0.07 | 0.01 |
|  | P | 0.02 | 0.44 | 0.00 | 0.28 | 0.31 | <0.001 | <0.001 | – | 0.04 | 0.48 | 0.93 |
| **EOS%** | r | 0.01 | 0.54*** | 0.37*** | 0.07 | 0.39*** | -0.01 | -0.19* | -0.19* | – | -0.17 | 0.30*** |
|  | P | 0.94 | <0.001 | <0.001 | 0.46 | <0.001 | 0.95 | 0.04 | 0.04 | – | 0.06 | <0.001 |
| **VitD** | r | 0.01 | -0.31** | -0.17 | 0.02 | -0.10 | -0.07 | 0.08 | 0.07 | -0.17 | – | -0.31*** |
|  | P | 0.96 | 0.01 | 0.06 | 0.80 | 0.29 | 0.44 | 0.40 | 0.48 | 0.06 | – | <0.001 |
| **Ferritin** | r | -0.04 | 0.29** | 0.29** | -0.02 | 0.23* | -0.08 | 0.01 | 0.01 | 0.30*** | -0.31*** | – |
|  | P | 0.67 | 0.00 | 0.00 | 0.83 | 0.01 | 0.38 | 0.94 | 0.93 | <0.001 | <0.001 | – |

Abbreviations: CRP, C-reactive protein; EOS, eosinophil count; Eo/LY, eosinophil-to-lymphocyte ratio; IL-6, interleukin-6; IgE, immunoglobulin E; LY, lymphocyte count; NLR, neutrophil-to-lymphocyte ratio; SII, systemic immune-inflammation index; EOS%, eosinophil percentage; VitD, vitamin D.
Correlation coefficients were calculated using Spearman’s rank correlation analysis.
*P < 0.05, **P < 0.01, ***P < 0.001.
